# An improved dual-indexing approach for multiplexed 16S rRNA gene sequencing on the Illumina MiSeq platform

**DOI:** 10.1186/2049-2618-2-6

**Published:** 2014-02-24

**Authors:** Douglas W Fadrosh, Bing Ma, Pawel Gajer, Naomi Sengamalay, Sandra Ott, Rebecca M Brotman, Jacques Ravel

**Affiliations:** 1 Institute for Genome Sciences, Department of Microbiology and Immunology, University of Maryland School of Medicine, 801 W. Baltimore Street, Baltimore, MD 21201, USA; 2 Institute for Genome Sciences, Department of Epidemiology and Public Health, University of Maryland School of Medicine, 801 W. Baltimore Street, Baltimore, MD 21201, USA

## Abstract

**Background:**

To take advantage of affordable high-throughput next-generation sequencing technologies to characterize microbial community composition often requires the development of improved methods to overcome technical limitations inherent to the sequencing platforms. Sequencing low sequence diversity libraries such as 16S rRNA amplicons has been problematic on the Illumina MiSeq platform and often generates sequences of suboptimal quality.

**Results:**

Here we present an improved dual-indexing amplification and sequencing approach to assess the composition of microbial communities from clinical samples using the V3-V4 region of the 16S rRNA gene on the Illumina MiSeq platform. We introduced a 0 to 7 bp “heterogeneity spacer” to the index sequence that allows an equal proportion of samples to be sequenced out of phase.

**Conclusions:**

Our approach yields high quality sequence data from 16S rRNA gene amplicons using both 250 bp and 300 bp paired-end MiSeq protocols and provides a flexible and cost-effective sequencing option.

## Background

The development of methods to detect fastidious or non-cultivable organisms through amplification and determination of the sequence of conserved genes, or culture-independent profiling, has precipitated a revolution in biology. It was recognized decades ago that the number of microbes seen on direct staining of environmental or human samples often exceeded by many orders of magnitude the number that could be cultured (termed "the great plate-count anomaly") [1]. Culture-independent profiling of bacterial communities relies on the amplification and sequencing of the generally considered universal 16S rRNA gene and has greatly increased appreciation for the complexity hidden in even seemingly simple microbial consortia. Advancements in next-generation sequencing technologies, in terms of throughput, sequence read length and accuracy, has had a major impact in the field by enabling large numbers of samples to be examined at greater depth. The Illumina MiSeq platform (San Diego, CA, USA) provides researchers with a scalable, high-throughput and streamlined sequencing platform to survey community composition from clinical and environmental samples. However, known limitations with the MiSeq platform associated with the sequencing of low sequence diversity samples has hampered harnessing its true potential to sequence 16S rRNA gene amplicons. The “low sequence diversity” issue arises in the first several cycles of a Miseq 16S rRNA gene amplicon sequencing run, during which successful cluster identification and phasing/pre-phasing calibration are dependent on heterogeneous base composition of targeted amplicons. Because of the nature of the 16S rRNA gene, amplicon pools are highly homogenous and are required to be co-sequenced with a heterogeneous control library, commonly phage PhiX, normally combined 1:1 with the amplicon pool. This improves the quality of the sequencing reads enough to yield a successful sequencing run (average quality value of 30 (Q30) >70%) but at the expense of having half of the sequence reads lost to a non-targeted template. Despite these limitations, the MiSeq sequencing platform’s high data yield and the 250 bp and 300 bp paired-end read (250PE and 300PE) protocols continue to be attractive to researchers. The technology enables the high resolution characterization of microbial communities with effective read lengths comparable to those obtained on the Roche/454 pyrosequencing platform (Branford, CT, USA) but for a fraction of the cost.

The most widely used 16S rRNA-based MiSeq sequencing strategies include a single- [2,3] or a recently developed dual-indexing [4] approach targeting the V4 hypervariable region of the 16S rRNA gene. These strategies leverage custom 16S rRNA PCR primers that enable multiplexing of samples and direct sequencing on the MiSeq instrument, but do not fully maximize the potential, or directly address the known limitations, of the sequencing technology. The single-indexing strategy requires large numbers of barcoded primers (one per sample) and custom sequencing primers, increasing costs and limiting flexibility. The current dual-indexing approach reduces the number of primers needed, but the low diversity issue still has not been addressed. This results in sequencing reads with bases of suboptimal quality that must be removed before analysis, resulting in shorter reads and analytical challenges. Here, we address the technical limitations of the MiSeq platform for 16S rRNA gene sequencing using both 250PE and 300PE protocols, and present a cost-effective approach to generate high-quality barcoded 16S rRNA gene amplicons by leveraging dual-indexed primers with built-in heterogeneity spacers.

## Methods and results

### V3-V4 amplification and sequencing strategy

The 16S rRNA gene consists of nine hypervariable regions flanked by regions of more conserved sequence. To maximize the effective length of the MiSeq’s 250PE and 300PE sequencing reads, a region of approximately 469 bp encompassing the V3 and V4 hypervariable regions of the 16S rRNA gene was targeted for sequencing. This region provides ample information for taxonomic classification of microbial communities from specimens associated with human microbiome studies and was used by the Human Microbiome Project [5], however, the approach described could be adapted to any primer pairs.

**Figure 1:**
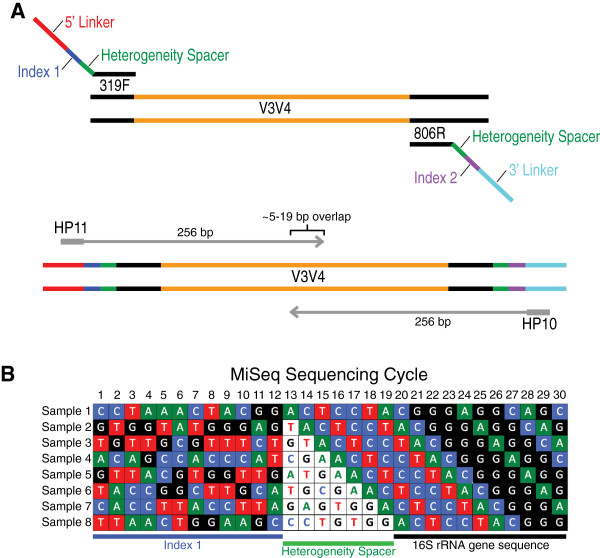
**Dual-indexed 16S rRNA gene PCR amplification strategy with heterogeneity spacer primers for sequencing on the MiSeq platform. (A)** Dual-indexed PCR amplification primers targeting the V3-V4 hypervariable regions of the 16S rRNA gene contain a heterogeneity spacer region and linker sequence optimized for sequencing on the Illumina MiSeq platform. Using this approach enables sequencing using the standard Illumina HP10 and HP11 sequencing primers allowing for additional sequencing flexibility. **(B)** Schematic showing the first thirty sequencing cycles of eight mock amplicons prepared using the dual-indexed approach. This diagram illustrates how the index sequence and heterogeneity spacer (colored letters, white background) helps to alleviate the “low sequence diversity” issue associated with the MiSeq platform by creating a more even base composition at each cycle of the run.

To amplify and sequence the V3-V4 hypervariable region of the 16S rRNA gene, primers were designed that contained: 1) a linker sequence allowing amplicons to bind to the flow cell and be sequenced using the standard Illumina HP10 or HP11 (Illumina, San Diego, CA, USA) sequencing primers; 2) a 12 bp index sequence; 3) a 0 to 7 bp “heterogeneity spacer” that we designed in this study to mitigate the issues caused by low sequence diversity amplicons (Additional file 1: Figure S1C); and 4) 16S rRNA gene universal primers (Figure 1A and Additional file 2). Genomic DNA extracted from clinical vaginal and anal swabs were amplified, normalized using the SequalPrep Normalization Kit (Life Technologies, Carlsbad, CA, USA) and pooled (11 pools with 271 to 426 samples per pool) prior to sequencing on the MiSeq platform (see Additional file 3 and Table 1 for number of samples per pools). The amplicon pools were prepared for sequencing with AMPure XT beads (Beckman Coulter Genomics, Danvers, MA, USA) and the size and quantity of the amplicon library were assessed on the LabChip GX (Perkin Elmer, Waltham, MA, USA) and with the Library Quantification Kit for Illumina (Kapa Biosciences, Woburn, MA, USA), respectively. PhiX Control library (v3) (Illumina) was combined with the amplicon library (expected at 20%). The library was clustered to a density of approximately 570 K/mm^2^. The libraries were sequenced either on 250PE or 300PE MiSeq runs and one library was sequenced with both protocols using the standard Illumina sequencing primers (Figure 1A), eliminating the need for a third (or fourth) index read. Sequencing data was available within approximately 48 hours. Image analysis, base calling and data quality assessment were performed on the MiSeq instrument.

**Table 1:** Raw sequence reads and post-QA/QC sequence reads statistics from nine 250PE and three 300PE MiSeq runs

**Run type**	**Pool ID (run ID)**	**Number of samples per pool**	**Raw sequence reads**						**QA/QC sequence reads**		
			**[DNA] (pM)**	**Cluster density**	**%phiX**	**% ≥Q30**	**Reported machine error**	**Number of read pairs**	**Total number of reads**	**Median read length**	**Mean read length**
				**(K/mm**^**2**^**)**							
									**(Forward; Reverse)**^**a**^	**(S1, S2)**^**a**^	**(S1, S2) ± SD**^**a**^
250PE	199–205 (130325)	352	5.5	509	16.3	87.4	1.24	9,413,692	5,994,627; 6,243,014	268; 256	266.04 ± 7.12; 246.99 ± 23.98
250PE	207–212 (130525)	387	6	583	14.4	86.3	1.32	10,656,207	7,438,345; 7,196,027	268; 259	265.41}8.02; 248.67}23.14
250PE	217–222 (130612)	337	6.2	650	15.1	84.1	1.5	11,706,459	7,806,262; 8,021,515	268; 244	265.03}8.48; 240.30}20.49
250PE	223–228 (130705)	364	5.5	839	10.7	84.6	1.24	13,398,346	9,703,996; 9,226,330	268; 261	265.00}8.84; 250.24}23.38
250PE	229–234 (130708)	366	4	625	8.4	81.1	1.57	11,071,952	8,089,983; 7,167,888	268; 232	265.85}6.94; 228.97}28.21
250PE	241–246 (130805)	305	12	527	8.9	92.5	1.34	9,615,279	7,393,672; 7,180,867	268; 264	266.21}6.23; 255.01}19.15
250PE	247–252 (130801)	299	9	429	17.3	91.2	1.07	7,787,755	5,267,816; 5,190,563	268; 266	266.39}7.73; 256.12}20.82
250PE	259–265 (130813)	426	7	445	9.3	90.7	1.46	7,919,928	5,920,074; 5,425,815	268; 265	265.10}9.83; 255.54}17.47
250PE	235–240 (130815)^b^	371	11	351	14.7	90.7	1.71	8,517,215	6,268,044; 5,848,845	268; 267	267.73}2.75; 262.44}13.29
300PE	235–240 (130916)^b^	371	11	514	13.9	90.9	1.44	12,522,115	9,328,369	499	497.19}6.93
300PE	266–270 (131011)	271	11	441	15.4	91.3	1.54	10,699,820	8,111,221	485	488.03}9.78
300PE	271–275 (131015)	276	12	592	12.3	90.4	1.52	14,241,937	10,912,710	495	491.05}10.56

250PE, 250 bp paired-end read; 300PE, 300 bp paired-end read; Q30, base call quality value >30; QA, quality assurance; QC, quality control; S1, sequence read 1; S2, Sequence read 2.

^a^300PE statistics for assembled paired-end sequences; ^b^This pool was sequenced with both 250PE and 300PE protocol.

### 250PE sequence data pre-processing

**Figure 2:**
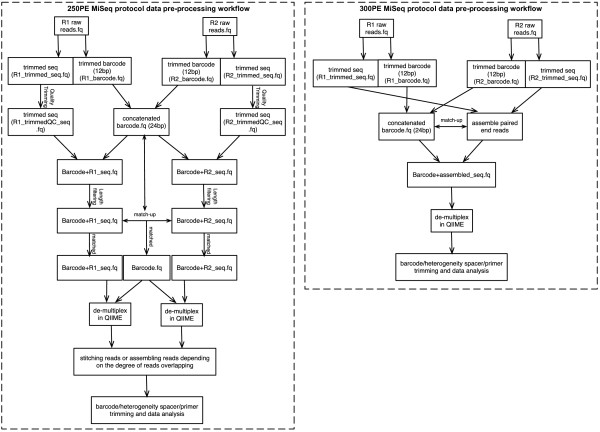
**Flow diagram outlining the sequence data analysis process.** Pre-processing for sequences generated with the 250 bp paired-end read (250PE; left panel) and 300 bp paired-end read (300PE; right panel) MiSeq protocols. R1 and R2 refers to read 1 and read 2.

A total of nine pools of amplicons were sequenced using the 250PE protocol, generating between 7.8 and 13.4 (mean 10.9) million total reads (Table 1, more detailed information listed in Additional file 4: Table S1). These high quality sequences (Additional file 1: Figure S1) were further processed using the procedures depicted in Figure 2A. Briefly, the index sequences contained in the first 12 bp of each paired-end read were extracted and concatenated to form a 24 bp dual-index barcode specific for each paired read and sample. Additional sequence read pre-processing included: 1) removal of primer sequence; 2) truncation of sequence reads not having an average quality of 20 over a 30 bp sliding window based on the phred algorithm [6,7] implemented previously [8,9]; and 3) removal of trimmed reads having less than 75% of their original length, as well as its paired read. These stringent criteria resulted in nearly 94% of reads being retained.

Further sequence reads processing was performed using QIIME (version 1.6.0,) [10] and included additional quality trimming, demultiplexing, and taxonomic assignments. QIIME quality trimming was performed using the following criteria: 1) truncate sequence reads before three consecutive low-quality bases and re-evaluate for length; 2) no ambiguous base calls; and 3) minimum sequence length of 150 bp after trimming. Between 5 and 10% of the reads were filtered out when applying these quality criteria. Paired-end reads were aligned to pre-aligned Greengene 16S rRNA gene sequences. If the two paired-end reads overlapped, the consensus sequence was generated; otherwise, they were simply stitched together. While stiching reads that do not overlap allows improved taxonomic assignments, it could introduce biases when calculating operational taxonomic units.

### 300PE sequence data pre-processing

A total of three pools of amplicons were sequenced using the 300PE MiSeq protocol, generating between 10.7 and 14.2 (mean 12.5) million total reads (Table 1). High-quality 300PEs (Additional file 5: Figure S2) were assembled as the reads were expected to overlap by approximately 90 bp. The analysis steps were similar to that used with the 250PE protocol (Figure 2B), with the exception that paired-end reads were assembled without preliminary quality trimming, using PANDAseq [11] and FLASH [12], as both perform error correction during assembly. Overall PANDAseq and FLASH yielded very similar results with 92.99% and 92.54% of the reads assembled, respectively. The final sequence length after barcode, heterogeneity spacer, and primer removal was 429 ± 7 bp and 429 ± 6.7 bp for PANDAseq and FLASH, respectively, with an average of approximately 11,600 reads per sample.

### Sequence data analysis

**Figure 3:**
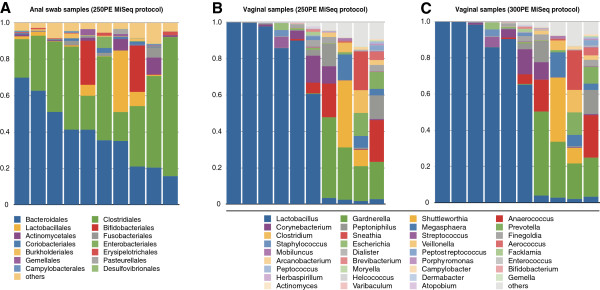
**Taxonomic assignments of clinical samples. (A)** Ten anal samples sequenced using 250 bp paired-end read (250PE) MiSeq protocol (pool 199–205). Ten vaginal samples sequenced using **(B)** 250PE and **(C)** 300 bp paired-end read (300PE) MiSeq protocols (pool 235–240) and analyzed using QIIME (version 1.6.0).

Concatenated 250 PE (420 to 440 bp long) and assembled 300PE reads were further processed, including denoising by clustering similar sequences with less than 3% dissimilarity using USEARCH [13] and *de novo* chimera detection, conducted with UCHIME v5.1 [14]. One of the libraries containing 371 samples (pool 235–240, Table 1 and Additional file 4: Table S1) was sequenced using both the 250PE and 300PE MiSeq protocol for further comparison. Taxonomic ranks were assigned to each sequence using Ribosomal Database Project (RDP) Naïve Bayesian Classifier v.2.2 [15] trained on the Greengenes database (Oct, 2012 version) [16], using 0.8 confidence values as cutoff. Taxonomic classification results for a subset of vaginal (from pool 235–240) and anal swabs (from pool 199–205) are shown in Figure 3 for both 250PE and 300PE MiSeq runs.

## Discussion

The MiSeq system provides a powerful sequencing platform for the rapid, high-throughput and in-depth characterization of microbial community composition using 16S rRNA gene amplicon sequencing. Its adoption was limited due to issues inherent to the technology. Two critical software processing steps for the generation of high-quality data on the MiSeq (cluster identification and phasing/prephasing rate determination) require a balanced base composition through the initial 12 to 18 cycles of the run. Because of this requirement, low sequence diversity 16S rRNA gene amplicon libraries do not sequence well on the MiSeq and the resulting data is of significantly lower overall quality than a more random library (that is, a metagenomic library). This problem is even further compounded in samples that are dominated by one or very few types of bacteria, as is the case with most vaginal microbial communities. Further, current methods for sequencing 16S rRNA gene amplicons on the MiSeq platform require a high proportion of PhiX Control library v3 (up to 50%) to be added to the amplicon library to modulate the overall sample base composition to help facilitate a successful sequencing run.

The dual-indexing amplification strategy, combined with the heterogeneity spacer design presented here, addresses the low sequence diversity issue by providing a much more balanced base composition through the entire duration of the sequencing run, increasing the overall quality of the sequence data (Additional file 1: Figure S1 and Additional file 5: Figure S2). In designing the primer system where the first 12 bases sequenced in each read are the in-line index, it is possible to select index combinations that ensure an equal proportion of each base throughout the first 12 cycles of the run. This approach also affords for multiplexing large numbers of samples at a reduced initial investment. Here, we are able to process up to 576 samples on a single sequencing run by multiplexing 24 forward and 24 reverse primers. The addition of the 0 to 7 bp heterogeneity spacer between the index sequence and the 16S rRNA sequence allows the 16S rRNA gene portion of the amplicons from an equal proportion of samples to be sequenced out of phase, further dampening the effect of the low sequence diversity issue of the MiSeq platform (Figure 1B and Additional file 1: Figure S1C). Furthermore, this feature makes it possible to dramatically reduce the ratio of PhiX Control library (v3) to amplicon library as the overall sample base composition is more even and yields data with higher overall average quality scores. Currently, the addition of as little as ~8% PhiX (Table 1) to an amplicon pool has been tested, indicating lower amounts of PhiX control library can be used to produce reads of comparable overall quality. This improved strategy produces higher quality reads (Table 1 and Additional file 4: Table S1) with substantially more usable reads, since less sequence space is dedicated to sequencing PhiX. Moreover, we have successfully adapted our method to both 250PE and 300PE MiSeq protocol, and the sequence read statistics shown in Table 1 and Additional file 1: Figure S1 and Additional file 5: Figure S2 indicates high yield and high quality sequencing reads generated for both protocols.

We designed two separate workflows that accommodate dual-indexed sequencing reads from either 250PE or 300PE protocols. We have applied a rather strict sequence quality filtering process because error rates on Illumina sequence tend to increase towards the end of the reads, with the second read being more affected than the first (see Additional file 1: Figure S1 and Additional file 5: Figure S2). This step improved the taxonomic assignment accuracy. Paired-end reads generated using the 250PE protocol can be either concatenated or stitched depending on the degree of sequence overlap. Paired-end reads generated using the 300PE protocol are assembled with great confidence using PANDAseq or FLASH. This assembly and base correction step substantially improves sequence quality as very few sequences did not pass the quality filter and no reads contained Ns (Additional file 4: Table S1). The purpose of both workflows is to maximize the information from paired-end reads to improve taxonomic assignment, while avoiding spurious paired-end reads assembly and allowing a strict, yet more flexible quality control. The taxonomic profiles of both 250PE and 300PE MiSeq runs (Figure 2) are highly similar, reflecting the high-quality sequence reads generated with either protocols and validating the method. That said, the dataset generated with the 300PE protocol has an approximate 90 bp overlap, making this protocol a preferred and superior approach that generates high-confidence paired-end assemblies compared to the 250PE, which at most overlap by 30 bp.

## Conclusion

Current methods for sequencing 16S rRNA gene amplicons on the MiSeq instrument use custom sequencing primers complementary to the universal 16S rRNA gene universal primer [2,4]. This custom setup creates potential problems when trying to multiplex samples on the same run that target different regions of the 16S rRNA gene. Our “heterogeneity spacer” approach to primer design and the use of the standard Illumina HP10 and HP11 sequencing primers, in combination with 300PE MiSeq protocol, yields highly reproducible datasets, and can be easily adapted to other 16 rRNA gene variable regions and future MiSeq protocol updates. While we have not observed potential amplification biases using different heterogeneity spacer length and/or sequence, it is possible that such bias exists.

## Availability of supporting data

All sequence data were deposited in SRA under BioProject PRJNA203369 (SRP023530, SRA082708). QIIME mapping files are provided in Additional file 6. The sequence processing scripts and their descriptions are available in Github (https://github.com/cwzkevin/MiSeq16S).

## Abbreviations

250PE: 250 base pair paired-end read; 300PE: 300 base pair paired-end read; bp: base pair; PCR: polymerase chain reaction; rRNA: ribosomal RNA.

## Competing interests

The authors declare that they have no competing interests.

## Authors’ contributions

JR, DWF, BM and RMB designed the study. DWF, SO and NS processed the samples and generated the sequence data. BM and PG analyzed the data. DWF, BM and JR interpreted the data. DF, BM and JR wrote the manuscript. All authors read and approved the final manuscript.

## Supplementary Material

Additional file 1: Figure S1 Quality and base composition assessment of a 250PE run. **(A)** Average quality plot of a dual-indexed 16S rRNA gene amplicon library sequenced on a paired-end 250PE MiSeq run, a cluster density of ~570, and a PhiX Control Library (v3) spike-in of ~20%. **(B)** Base composition plot of the 250PE MiSeq run from (A). **(C)** Base composition plot from a 250PE MiSeq run prepared from a 16S rRNA gene amplicon pool that employed the strategy described by Caporaso and colleagues [2]. Click here for file

Additional file 2 Primer sequences. Click here for file

Additional file 3 Supplementary methods. Click here for file

Additional file 4: Table S1 Detailed raw sequence reads statistics and post-QA/QC sequence reads statistics from nine 250PE and three 300PE MiSeq runs. Click here for file

Additional file 5: Figure S2 Quality and base composition assessment of a 300PE run. **(A)** Average quality plot of a dual-indexed 16S rRNA gene amplicon library sequenced on a paired-end 300PE MiSeq run, a cluster density of ~570, and a PhiX Control Library (v3) spike-in of ~20%. **(B)** Base composition plot of the 300PE MiSeq run from (A). Click here for file

Additional file 6 QIIME mapping files associated with each sequencing run. Click here for file
