# Supplementary figures and images for: An improved dual-indexing approach for multiplexed 16S rRNA gene sequencing on the Illumina MiSeq platform
Source: Microbiome. 2014 Feb 24;2:6. doi: 10.1186/2049-2618-2-6 (PMC3940169; doi:10.1186/2049-2618-2-6)

**A.**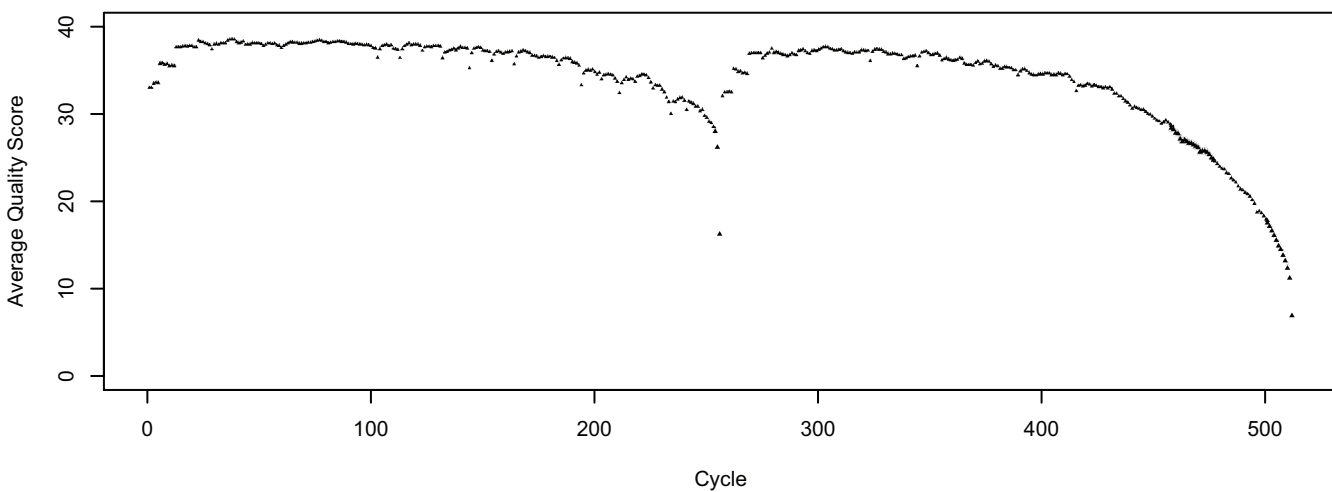**B.**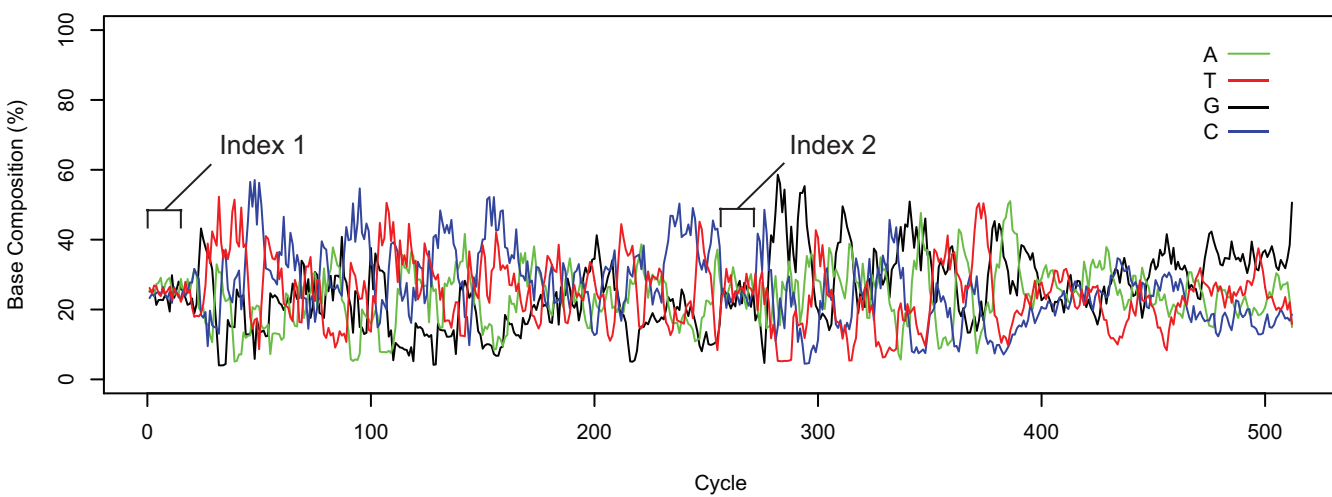**C.**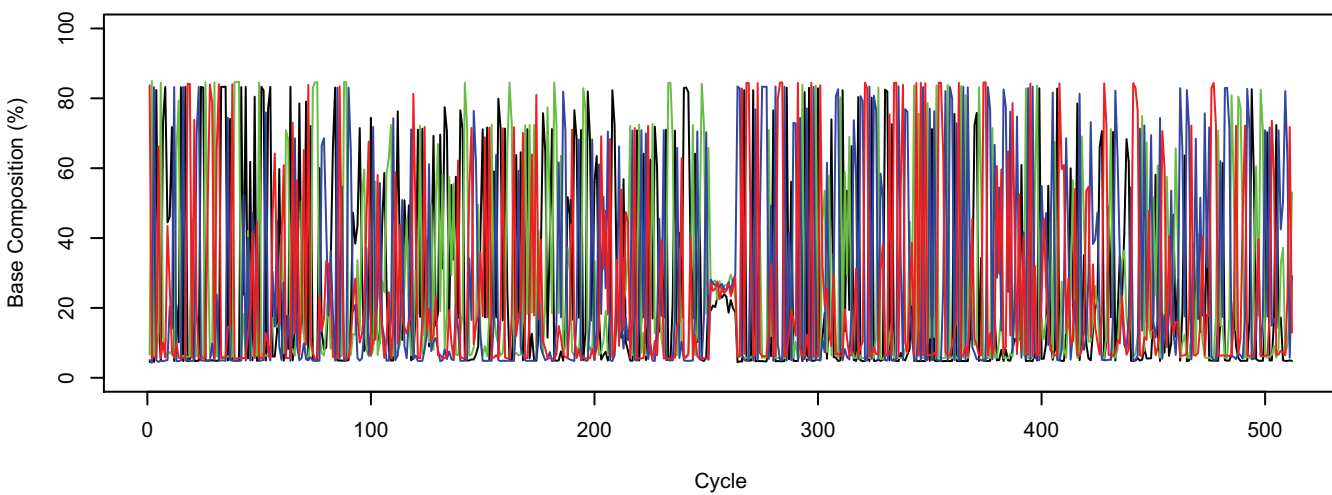

Supplement: Additional file 1: Figure S1 — Quality and base composition assessment of a 250PE run. (A) Average quality plot of a dual-indexed 16S rRNA gene amplicon library sequenced on a paired-end 250PE MiSeq run, a cluster density of ~570, and a PhiX Control Library (v3) spike-in of ~20%. (B) Base composition plot of the 250PE MiSeq run from (A). (C) Base composition plot from a 250PE MiSeq run prepared from a 16S rRNA gene amplicon pool that employed the strategy described by Caporaso and colleagues [2]. [file 2049-2618-2-6-S1.pdf]

**A.**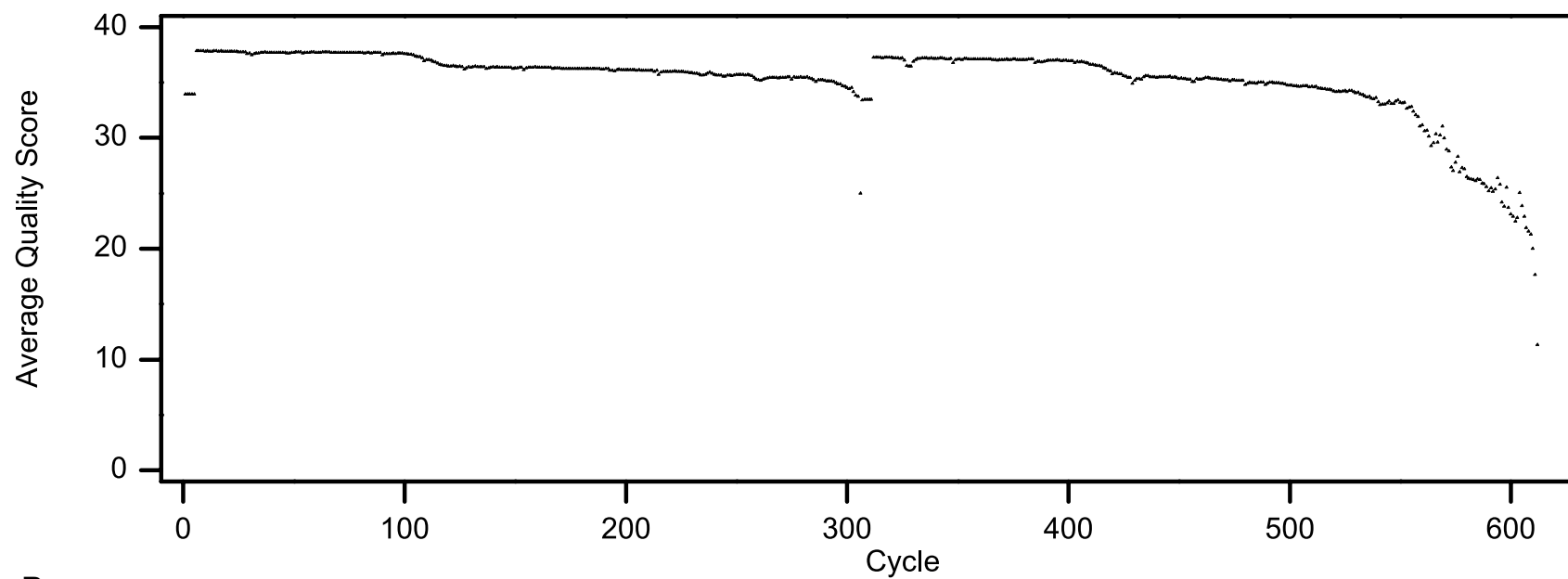**B.**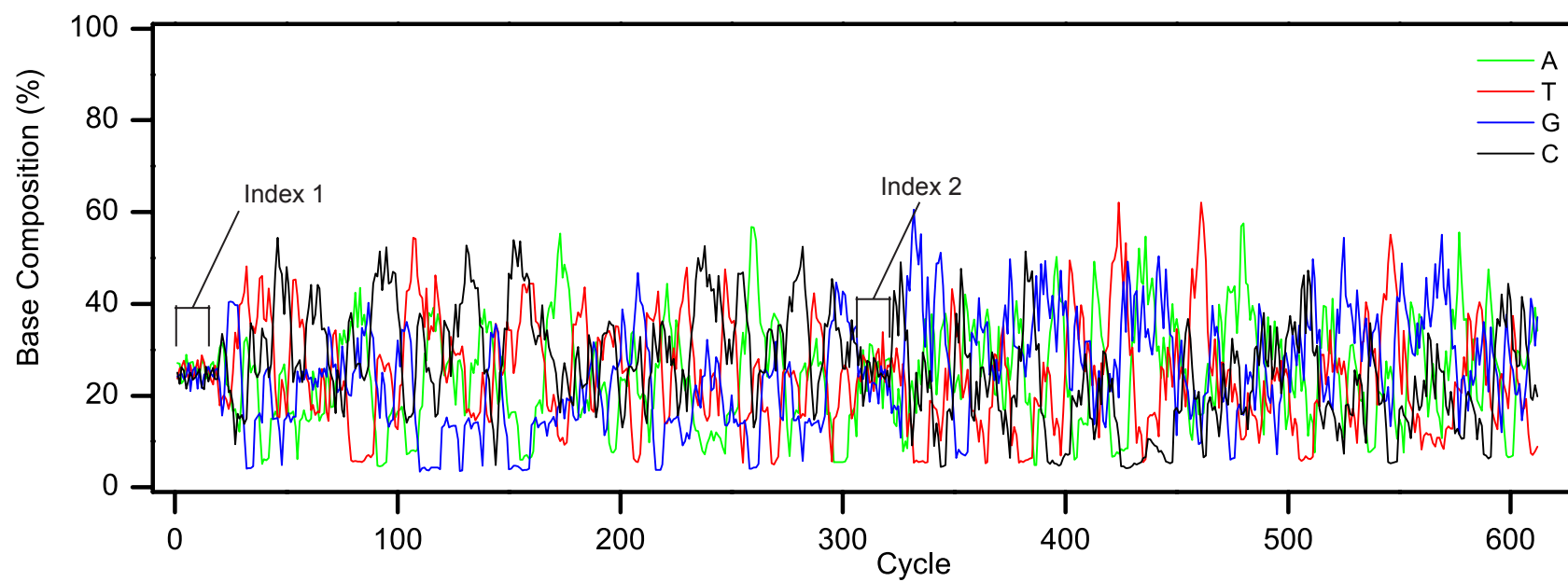

Supplement: Additional file 5: Figure S2 — Quality and base composition assessment of a 300PE run. (A) Average quality plot of a dual-indexed 16S rRNA gene amplicon library sequenced on a paired-end 300PE MiSeq run, a cluster density of ~570, and a PhiX Control Library (v3) spike-in of ~20%. (B) Base composition plot of the 300PE MiSeq run from (A). [file 2049-2618-2-6-S5.pdf]
