# Supplementary material for: An improved dual-indexing approach for multiplexed 16S rRNA gene sequencing on the Illumina MiSeq platform
Source: Microbiome. 2014 Feb 24;2:6. doi: 10.1186/2049-2618-2-6 (PMC3940169; doi:10.1186/2049-2618-2-6)
Supplement: Additional file 3 — Supplementary methods. [file 2049-2618-2-6-S3.docx]

1. Protocol Aim
   1. This protocol describes the workflow used for the amplification, normalization and pooling of 16s rRNA PCR amplicons prior to sequencing on the Illumina MiSeq using the 319F/806R dual-indexed PCR approach.
2. Prior to PCR Amplification
   1. Reconstitute dual-indexed 319F and 806R primers to 100μM using 1x TE Buffer. Dilute indexed primers to 1 μM with sterile water and array into six 96-well plates according to Appendix 1 at the end of this document.
   2. Extract and quantitate gDNA template. Dilute gDNA to 5 ng/μl and array into a 96-well plate.
3. PCR Amplification
   1. To process up to 96 samples at one time, a master mix can be made containing 12.5 μl of Phusion High-Fidelity PCR Master Mix with HF Buffer, 0.75 μl DMSO, and 1.75 μl of sterile water per reaction. Using a multichannel pipette, dispense 15 μl of this master mix solution into each well of a 96-well plate. A No Template Control Plate should prepared in the same way with each sample plate using water instead of gDNA template.
   2. Using the ViaFlo96 (or a multichannel pipette) transfer 5 μl of primer mix into each well of the 96-well plate making sure to match the well of the primer plate to the well of the PCR plate.
   3. Using the ViaFlo96 (or a multichannel pipette) transfer 5 μl of gDNA template to each well of a 96-well plate.
   4. Seal the 96-well plate with a clear film strip, vortex briefly and centrifuge the plate to collect liquid at the bottom of the plate.
   5. A No Template Control (NTC) Plate should prepared in the same way using sterile water in place of gDNA template.
   6. Place in a thermocycler and cycle at:

98C 30s

98C 15s \

58C 15s 30 x

72C 15s /

72C 60s

4C hold

1. Gel Electrophoresis
   1. Remove 96 well plate from thermocycler and centrifuge to collect liquid at the bottom of the wells.
   2. In a new 96-well plate combine 18 μl of sterile water and 2 μl of PCR product and load 20 μl onto a 2% E-gel 96.
   3. Run E-gel for approximately 5 minutes or until the PCR products run far enough into the gel to confirm that the PCR was successful.
   4. Repeat for the NTC plate to confirm that there was no contamination.
2. Normalization and Pooling

The SequalPrep Normalization Kit is used to normalize samples.

- 1. Using the ViaFlo96 (or a multichannel pipette) add 20 μl of Binding Buffer to the SequalPrep plate.
  2. Using the ViaFlo96 (or a multichannel pipette) transfer 20 μl of PCR product from the PCR plate to the corresponding well of the SequalPrep plate.
  3. Seal SequalPrep Plate, vortex, and centrifuge to collect all liquid at the bottom of the wells.
  4. Incubate on the bench top for at least 1 hour.
  5. Using the ViaFlo96 (or a multichannel pipette) aspirate the liquid from the wells and discard. Be sure not to contact the side of the wells.
  6. Add 50 μl of Wash Buffer to each well using the ViaFlo96 (or multichannel pipette) and pipette up and down slowly 5 times. Aspirate liquid from wells and discard.
  7. Add 20 μl of Elution Buffer to each well. Seal plate, vortex and centrifuge to collect liquid at the bottom of the wells.
  8. Incubate plate at room temperature for 10 minutes.
  9. Add 15 μl of each normalized sample that had a positive PCR amplification to a 2ml tube.

Appendix #1 – Dual Index Primer Plate Formats

|  | Forward Primer - Dual Index Primer Plate 1 | | | | | | | | | | | |  |  | Reverse Primer - Dual Index Primer Plate 1 | | | | | | | | | | | |
| --- | --- | --- | --- | --- | --- | --- | --- | --- | --- | --- | --- | --- | --- | --- | --- | --- | --- | --- | --- | --- | --- | --- | --- | --- | --- | --- |
| A | 1 | 2 | 3 | 4 | 5 | 6 | 7 | 8 | 9 | 10 | 11 | 12 |  | A | 1 | 2 | 3 | 4 | 5 | 6 | 7 | 8 | 9 | 10 | 11 | 12 |
| B | 13 | 14 | 15 | 16 | 17 | 18 | 19 | 20 | 21 | 22 | 23 | 24 |  | B | 13 | 14 | 15 | 16 | 17 | 18 | 19 | 20 | 21 | 22 | 23 | 24 |
| C | 1 | 2 | 3 | 4 | 5 | 6 | 7 | 8 | 9 | 10 | 11 | 12 |  | C | 2 | 3 | 4 | 5 | 6 | 7 | 8 | 9 | 10 | 11 | 12 | 13 |
| D | 13 | 14 | 15 | 16 | 17 | 18 | 19 | 20 | 21 | 22 | 23 | 24 |  | D | 14 | 15 | 16 | 17 | 18 | 19 | 20 | 21 | 22 | 23 | 24 | 1 |
| E | 1 | 2 | 3 | 4 | 5 | 6 | 7 | 8 | 9 | 10 | 11 | 12 |  | E | 3 | 4 | 5 | 6 | 7 | 8 | 9 | 10 | 11 | 12 | 13 | 14 |
| F | 13 | 14 | 15 | 16 | 17 | 18 | 19 | 20 | 21 | 22 | 23 | 24 |  | F | 15 | 16 | 17 | 18 | 19 | 20 | 21 | 22 | 23 | 24 | 1 | 2 |
| G | 1 | 2 | 3 | 4 | 5 | 6 | 7 | 8 | 9 | 10 | 11 | 12 |  | G | 4 | 5 | 6 | 7 | 8 | 9 | 10 | 11 | 12 | 13 | 14 | 15 |
| H | 13 | 14 | 15 | 16 | 17 | 18 | 19 | 20 | 21 | 22 | 23 | 24 |  | H | 16 | 17 | 18 | 19 | 20 | 21 | 22 | 23 | 24 | 1 | 2 | 3 |
|  | Forward Primer - Dual Index Primer Plate 2 | | | | | | | | | | | |  |  | Reverse Primer - Dual Index Primer Plate 2 | | | | | | | | | | | |
| A | 1 | 2 | 3 | 4 | 5 | 6 | 7 | 8 | 9 | 10 | 11 | 12 |  | A | 5 | 6 | 7 | 8 | 9 | 10 | 11 | 12 | 13 | 14 | 15 | 16 |
| B | 13 | 14 | 15 | 16 | 17 | 18 | 19 | 20 | 21 | 22 | 23 | 24 |  | B | 17 | 18 | 19 | 20 | 21 | 22 | 23 | 24 | 1 | 2 | 3 | 4 |
| C | 1 | 2 | 3 | 4 | 5 | 6 | 7 | 8 | 9 | 10 | 11 | 12 |  | C | 6 | 7 | 8 | 9 | 10 | 11 | 12 | 13 | 14 | 15 | 16 | 17 |
| D | 13 | 14 | 15 | 16 | 17 | 18 | 19 | 20 | 21 | 22 | 23 | 24 |  | D | 18 | 19 | 20 | 21 | 22 | 23 | 24 | 1 | 2 | 3 | 4 | 5 |
| E | 1 | 2 | 3 | 4 | 5 | 6 | 7 | 8 | 9 | 10 | 11 | 12 |  | E | 7 | 8 | 9 | 10 | 11 | 12 | 13 | 14 | 15 | 16 | 17 | 18 |
| F | 13 | 14 | 15 | 16 | 17 | 18 | 19 | 20 | 21 | 22 | 23 | 24 |  | F | 19 | 20 | 21 | 22 | 23 | 24 | 1 | 2 | 3 | 4 | 5 | 6 |
| G | 1 | 2 | 3 | 4 | 5 | 6 | 7 | 8 | 9 | 10 | 11 | 12 |  | G | 8 | 9 | 10 | 11 | 12 | 13 | 14 | 15 | 16 | 17 | 18 | 19 |
| H | 13 | 14 | 15 | 16 | 17 | 18 | 19 | 20 | 21 | 22 | 23 | 24 |  | H | 20 | 21 | 22 | 23 | 24 | 1 | 2 | 3 | 4 | 5 | 6 | 7 |
|  | Forward Primer - Dual Index Primer Plate 3 | | | | | | | | | | | |  |  | Reverse Primer - Dual Index Primer Plate 3 | | | | | | | | | | | |
| A | 1 | 2 | 3 | 4 | 5 | 6 | 7 | 8 | 9 | 10 | 11 | 12 |  | A | 9 | 10 | 11 | 12 | 13 | 14 | 15 | 16 | 17 | 18 | 19 | 20 |
| B | 13 | 14 | 15 | 16 | 17 | 18 | 19 | 20 | 21 | 22 | 23 | 24 |  | B | 21 | 22 | 23 | 24 | 1 | 2 | 3 | 4 | 5 | 6 | 7 | 8 |
| C | 1 | 2 | 3 | 4 | 5 | 6 | 7 | 8 | 9 | 10 | 11 | 12 |  | C | 10 | 11 | 12 | 13 | 14 | 15 | 16 | 17 | 18 | 19 | 20 | 21 |
| D | 13 | 14 | 15 | 16 | 17 | 18 | 19 | 20 | 21 | 22 | 23 | 24 |  | D | 22 | 23 | 24 | 1 | 2 | 3 | 4 | 5 | 6 | 7 | 8 | 9 |
| E | 1 | 2 | 3 | 4 | 5 | 6 | 7 | 8 | 9 | 10 | 11 | 12 |  | E | 11 | 12 | 13 | 14 | 15 | 16 | 17 | 18 | 19 | 20 | 21 | 22 |
| F | 13 | 14 | 15 | 16 | 17 | 18 | 19 | 20 | 21 | 22 | 23 | 24 |  | F | 23 | 24 | 1 | 2 | 3 | 4 | 5 | 6 | 7 | 8 | 9 | 10 |
| G | 1 | 2 | 3 | 4 | 5 | 6 | 7 | 8 | 9 | 10 | 11 | 12 |  | G | 12 | 13 | 14 | 15 | 16 | 17 | 18 | 19 | 20 | 21 | 22 | 23 |
| H | 13 | 14 | 15 | 16 | 17 | 18 | 19 | 20 | 21 | 22 | 23 | 24 |  | H | 24 | 1 | 2 | 3 | 4 | 5 | 6 | 7 | 8 | 9 | 10 | 11 |
|  | Forward Primer - Dual Index Primer Plate 4 | | | | | | | | | | | |  |  | Reverse Primer - Dual Index Primer Plate 4 | | | | | | | | | | | |
| A | 1 | 2 | 3 | 4 | 5 | 6 | 7 | 8 | 9 | 10 | 11 | 12 |  | A | 13 | 14 | 15 | 16 | 17 | 18 | 19 | 20 | 21 | 22 | 23 | 24 |
| B | 13 | 14 | 15 | 16 | 17 | 18 | 19 | 20 | 21 | 22 | 23 | 24 |  | B | 1 | 2 | 3 | 4 | 5 | 6 | 7 | 8 | 9 | 10 | 11 | 12 |
| C | 1 | 2 | 3 | 4 | 5 | 6 | 7 | 8 | 9 | 10 | 11 | 12 |  | C | 14 | 15 | 16 | 17 | 18 | 19 | 20 | 21 | 22 | 23 | 24 | 1 |
| D | 13 | 14 | 15 | 16 | 17 | 18 | 19 | 20 | 21 | 22 | 23 | 24 |  | D | 2 | 3 | 4 | 5 | 6 | 7 | 8 | 9 | 10 | 11 | 12 | 13 |
| E | 1 | 2 | 3 | 4 | 5 | 6 | 7 | 8 | 9 | 10 | 11 | 12 |  | E | 15 | 16 | 17 | 18 | 19 | 20 | 21 | 22 | 23 | 24 | 1 | 2 |
| F | 13 | 14 | 15 | 16 | 17 | 18 | 19 | 20 | 21 | 22 | 23 | 24 |  | F | 3 | 4 | 5 | 6 | 7 | 8 | 9 | 10 | 11 | 12 | 13 | 14 |
| G | 1 | 2 | 3 | 4 | 5 | 6 | 7 | 8 | 9 | 10 | 11 | 12 |  | G | 16 | 17 | 18 | 19 | 20 | 21 | 22 | 23 | 24 | 1 | 2 | 3 |
| H | 13 | 14 | 15 | 16 | 17 | 18 | 19 | 20 | 21 | 22 | 23 | 24 |  | H | 4 | 5 | 6 | 7 | 8 | 9 | 10 | 11 | 12 | 13 | 14 | 15 |
|  | Forward Primer - Dual Index Primer Plate 5 | | | | | | | | | | | |  |  | Reverse Primer - Dual Index Primer Plate 5 | | | | | | | | | | | |
| A | 1 | 2 | 3 | 4 | 5 | 6 | 7 | 8 | 9 | 10 | 11 | 12 |  | A | 17 | 18 | 19 | 20 | 21 | 22 | 23 | 24 | 1 | 2 | 3 | 4 |
| B | 13 | 14 | 15 | 16 | 17 | 18 | 19 | 20 | 21 | 22 | 23 | 24 |  | B | 5 | 6 | 7 | 8 | 9 | 10 | 11 | 12 | 13 | 14 | 15 | 16 |
| C | 1 | 2 | 3 | 4 | 5 | 6 | 7 | 8 | 9 | 10 | 11 | 12 |  | C | 18 | 19 | 20 | 21 | 22 | 23 | 24 | 1 | 2 | 3 | 4 | 5 |
| D | 13 | 14 | 15 | 16 | 17 | 18 | 19 | 20 | 21 | 22 | 23 | 24 |  | D | 6 | 7 | 8 | 9 | 10 | 11 | 12 | 13 | 14 | 15 | 16 | 17 |
| E | 1 | 2 | 3 | 4 | 5 | 6 | 7 | 8 | 9 | 10 | 11 | 12 |  | E | 19 | 20 | 21 | 22 | 23 | 24 | 1 | 2 | 3 | 4 | 5 | 6 |
| F | 13 | 14 | 15 | 16 | 17 | 18 | 19 | 20 | 21 | 22 | 23 | 24 |  | F | 7 | 8 | 9 | 10 | 11 | 12 | 13 | 14 | 15 | 16 | 17 | 18 |
| G | 1 | 2 | 3 | 4 | 5 | 6 | 7 | 8 | 9 | 10 | 11 | 12 |  | G | 20 | 21 | 22 | 23 | 24 | 1 | 2 | 3 | 4 | 5 | 6 | 7 |
| H | 13 | 14 | 15 | 16 | 17 | 18 | 19 | 20 | 21 | 22 | 23 | 24 |  | H | 8 | 9 | 10 | 11 | 12 | 13 | 14 | 15 | 16 | 17 | 18 | 19 |
|  | Forward Primer - Dual Index Primer Plate 6 | | | | | | | | | | | |  |  | Reverse Primer - Dual Index Primer Plate 6 | | | | | | | | | | | |
| A | 1 | 2 | 3 | 4 | 5 | 6 | 7 | 8 | 9 | 10 | 11 | 12 |  | A | 21 | 22 | 23 | 24 | 1 | 2 | 3 | 4 | 5 | 6 | 7 | 8 |
| B | 13 | 14 | 15 | 16 | 17 | 18 | 19 | 20 | 21 | 22 | 23 | 24 |  | B | 9 | 10 | 11 | 12 | 13 | 14 | 15 | 16 | 17 | 18 | 19 | 20 |
| C | 1 | 2 | 3 | 4 | 5 | 6 | 7 | 8 | 9 | 10 | 11 | 12 |  | C | 22 | 23 | 24 | 1 | 2 | 3 | 4 | 5 | 6 | 7 | 8 | 9 |
| D | 13 | 14 | 15 | 16 | 17 | 18 | 19 | 20 | 21 | 22 | 23 | 24 |  | D | 10 | 11 | 12 | 13 | 14 | 15 | 16 | 17 | 18 | 19 | 20 | 21 |
| E | 1 | 2 | 3 | 4 | 5 | 6 | 7 | 8 | 9 | 10 | 11 | 12 |  | E | 23 | 24 | 1 | 2 | 3 | 4 | 5 | 6 | 7 | 8 | 9 | 10 |
| F | 13 | 14 | 15 | 16 | 17 | 18 | 19 | 20 | 21 | 22 | 23 | 24 |  | F | 11 | 12 | 13 | 14 | 15 | 16 | 17 | 18 | 19 | 20 | 21 | 22 |
| G | 1 | 2 | 3 | 4 | 5 | 6 | 7 | 8 | 9 | 10 | 11 | 12 |  | G | 24 | 1 | 2 | 3 | 4 | 5 | 6 | 7 | 8 | 9 | 10 | 11 |
| H | 13 | 14 | 15 | 16 | 17 | 18 | 19 | 20 | 21 | 22 | 23 | 24 |  | H | 12 | 13 | 14 | 15 | 16 | 17 | 18 | 19 | 20 | 21 | 22 | 23 |
